# Interaction between polymorphisms in cell-cycle genes and environmental factors in regulating cholinesterase activity in people with exposure to omethoate

**DOI:** 10.1098/rsos.172357

**Published:** 2018-05-16

**Authors:** Xiaoran Duan, Yongli Yang, Sihua Wang, Xiaolei Feng, Tuanwei Wang, Pengpeng Wang, Wu Yao, Liuxin Cui, Wei Wang

**Affiliations:** 1 Department of Occupational and Environmental Health, College of Public Health, Zhengzhou University, Zhengzhou, People's Republic of China; 2 Department of Epidemiology and Biostatistics, College of Public Health, Zhengzhou University, Zhengzhou, People's Republic of China; 3 Department of Occupational Health, Henan Institute for Occupational Medicine, Zhengzhou, People's Republic of China

**Keywords:** generalized multifactor dimensionality reduction, omethoate, cholinesterase activity, single nucleotide polymorphisms, interaction

## Abstract

Cholinesterase activity (ChA), the effective biomarker for organophosphate pesticide exposure, is possibly affected by single nucleotide polymorphisms (SNPs) in cell-cycle-related genes. One hundred and eighty workers with long-term exposure to omethoate and 115 healthy controls were recruited to explore the gene–gene and gene–environment interactions. The acetylthiocholine and dithio-bis-(nitrobenzoic acid) method was used to detect the cholinesterase activities in whole blood, erythrocytes and plasma. Genetic polymorphisms were determined by the PCR-RFLP and direct PCR electrophoresis methods. Statistical results showed that the cholinesterase activities of whole blood, erythrocytes and plasma in the exposure group were significantly lower than those in the control group (*p *< 0.001), and erythrocyte cholinesterase activities were associated with gender, smoking and drinking in the exposure group (*p *< 0.05). Single-locus analyses showed that there is a statistically significant difference in the ChA among the genotypes CC, CA and AA of the *p21* rs1801270 locus in the control group (*p *= 0.033), but not in the exposure group. A significant interaction between genes and environmental factors (i.e. *p53*, *p21*, *mdm2,* gender, smoking and drinking) affecting ChA was found through a generalized multifactor dimensionality reduction analysis. These obtained markers will be useful in further marker-assisted selection in workers with exposure to omethoate.

## 1. Introduction

Omethoate is a broad category of organophosphorous pesticides (OPs) with increasing domestic use, which has highly toxic and effective features [1]. The main cause of toxicity to humans is the targeting of acetylcholinesterase (AChE), which hydrolyses acetylcholine (ACh) in the cholinergic synapses and in neuromuscular junctions where this enzyme plays a key role in cell-to-cell communication [2]. Its acute toxicity mechanism is mainly dependent on the inhibition of cholinesterase (ChE) activity, leading to the accumulation of ACh *in vivo*, thus stimulating muscarinic and nicotinic receptors to cause neurological disorders. Cholinesterase activity (ChA) has served as an organophosphate pesticide-exposure biomarker [3,4].

Recently, the potential genotoxicity of omethoate has attracted wide attention, causing chromosomal DNA damage including alkylation lesions, single-strand breaks and micronucleus [5–7]. Genetic polymorphisms in cell-cycle-regulating genes may influence the DNA damage [8]. It has been shown that DNA damage could change the level of ChA through effects on the quantity and quality of red blood cells. When cells are damaged, cell division will stagnate in the G1 phase of mitosis, so the checkpoint is extremely important in this phase [9]. In cell-cycle checkpoint regulation, the arrest of the cell cycle in G1/S transition is a p53-dependent process [10]. The downstream gene of the *p53* gene, *p21*, can prevent cell-cycle progression in the G1/S and G2/M phases and plays an important role in suppressing cancer [11]. The *Mdm2* gene is a negative regulator of *p53* which inhibits *p53* expression [12]; and *p53* could upregulate *p21* expression in response to DNA-damaging agents [13]. Many studies have shown that rs1042522, rs17878362, rs1625895, rs1801270 and rs1059234 for the *p*53 and *p21* genes have a close relationship with cancers [14–20]. *Mdm2* gene rs3730485 was also intimately related to tumour occurrence [21]. So far, no study has been found related to the correlations between the polymorphisms of *p53*, *p21* and *mdm2* genes and cholinesterase activities.

However, a single polymorphism in a particular gene is unlikely to explain completely the variability determined in ChA. With the in-depth development of disease genetics research, it has been found that the effects of genes on disease are very complex, and could be the result of the interaction between multiple genes and environmental factors [22–24]. In this study, polymorphisms in cell-cycle-regulating gene pathways were analysed to determine whether the gene polymorphisms are associated with response to OP exposure. Also, the interactions between polymorphisms and environmental factors were analysed to obtain a better understanding of the damage response to OP exposure.

Generalized multifactor dimensionality reduction (GMDR) analysis is customarily used to evaluate the higher order gene–gene and gene–environment interactions underlying a complex trait [25]. Compared to the available methods, our proposed method has several major improvements, including allowing for covariate adjustments, missing marker genotypes, and dichotomous and continuous phenotypes [26]. Therefore, we aimed to explore the gene–gene and gene–environment interactions and their effects on cholinesterase activities using the GMDR method, which can provide more insights into the genetic background for OP toxicity research and biological monitoring of occupational exposure.

## 2. Material and methods

### 2.1. Study population

One hundred and eighty workers and 115 healthy persons were recruited as the research population. Workers exposed to omethoate for longer than 8 years were included in the exposure group; for the control group, the inclusion criteria were the non-exposure to toxicants, and belonging to the same region and similar social class. For the exposure population, type of work includes packing, screwing, filling and corking work. The concentration of pesticides in the working environment was detected by specialized health institutions every year, and found to be lower than occupational exposure limits prescribed by the state. Professional investigators and doctors collected their occupational history, basic situation and biological samples. Individual smoking status was divided into smoking and non-smoking; smoking refers to persons who smoked at least one cigarette a day for at least 1 year consecutively or cumulatively during their lifetime. Drinking refers to persons who drank alcohol once a week or more for at least 1 year consecutively. Before conducting the study, approval was obtained from the Zhengzhou University Ethics Committee and the approved informed consent form was signed by each subject.

### 2.2. Research methods

#### 2.2.1. The detection of genetic polymorphisms

Genomic DNA from peripheral blood was extracted in strict accordance with the Blood DNA Kit (BioTeke Corporation, Beijing, China) and to determine DNA purity and concentration. Six polymorphic loci of *p53* rs1042522, *p53* rs17878362, *p53* rs1625895, *p21* rs1801270, *p21* rs1059234 and *mdm2* rs3730485 from the three genes were detected using genomic DNA isolated from peripheral blood lymphocytes. For the detection method for genotyping, primer sequences and restriction endonucleases, the reader is referred to a previous publication by our group [9].

#### 2.2.2. The determination of cholinesterase activity

Cholinesterase activity was determined in strict accordance with the occupational health standards of the People's Republic of China (GBZ 52–2002). The acetylthiocholine and dithio-bis-(nitrobenzoic acid) method was adopted to detect the cholinesterase activities in whole blood, erythrocytes and plasma. The specific principle is as follows: acetylthiocholine is hydrolysed to thiocholine and acetate under the action of cholinesterase. Quantitative analysis is performed of the yellow compound formed by thiocholine and dithio-bis-(nitrobenzoic acid). The amount of thiocholine can reflect the value of ChA. The measured absorbance of whole blood and plasma was substituted into the following formula to calculate the whole blood and plasma cholinesterase activities using the acetylthiocholine and dithio-bis-(nitrobenzoic acid) method. The subtraction of plasma ChA from whole blood ChA gives erythrocyte ChA.

$$\frac{\text{Absorbance}}{1.36\times 10^{4}}\times 10^{3}\times 2.6\times \frac{1}{6}\times \frac{10^{3}}{10}\times \frac{10}{4}=\text{Absorbance}\times 7.97.$$

1 µM thiocholine produced per millilitre of whole blood (plasma or red blood cells) per minute was regarded as one unit of ChA. In the formula, 1.36 × 10^4^ represents the absorbance of nitrobenzoate ions formed in the enzyme assay. The number of nitrobenzoate ions is the same as that of thiocholine.

### 2.3. Statistical analysis

The data were analysed using the SPSS21.0 software. Methods of representation and examination were based on the distribution of quantitative data. The effects of genetic polymorphisms on ChA were analysed using one-way analysis of variance or two independent sample *t*-test after merging the genotypes, and the Dunnett method was used to perform the comparisons between the two groups. The GMDR software was used to detect gene–gene and gene–environment interactions under various scenarios. All statistical tests were two sided, and the level of statistical significance was set at *α *= 0.05.

## 3. Results

### 3.1. Population-based data

The compared results of gender, age, smoking and drinking between the two groups are presented in table 1. The differences have statistical significance in the gender, age, smoking and drinking characteristics between the two groups.

**Table 1.** General characteristics of exposure and control groups. To compare the two groups, two independent sample *t*-test was adopted for the difference of age; the remaining variables were analysed with the *χ*^2^-test.

variable	exposure (*n* = 180)	controls (*n* = 115)	*χ^2^*/*t*	*p*-value
age (years)	43.79 ± 8.01	38.89 ± 8.25	5.069	<0.001
gender				
male	137	54	26.130	<0.001
female	43	61		
smoking				
yes	63	12	22.333	<0.001
no	117	103		
drinking				
yes	16	30	15.769	<0.001
no	164	85		

### 3.2. The detection of external exposure

According to the detection reports from 2011 to 2013, the 8 h time-weighted average and short-term exposure concentrations were lower than occupational exposure limits prescribed by the state in each type of work.

### 3.3. The determination of cholinesterase activity

**Figure 1.**
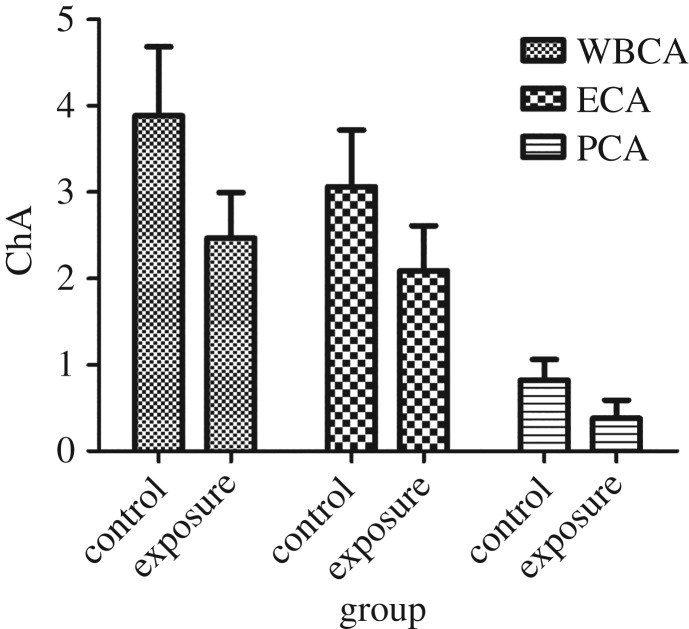
Comparison of the cholinesterase activities between the exposure group and the control group. The data are represented as $\bar{x}\pm s$. WBCA, whole blood cholinesterase activity; ECA, erythrocyte cholinesterase activity; PCA, plasma cholinesterase activity.

Figure 1 shows the results of cholinesterase activity measurement in the two groups. The results of *t*-tests showed that the cholinesterase activities of whole blood, erythrocytes and plasma in the exposure group were lower than those in the control group (2.47 ± 0.53 versus 3.89 ± 0.80, 2.09 ± 0.52 versus 3.06 ± 0.65, 0.38 ± 0.21 versus 0.82 ± 0.24, *p *< 0.001). The three sets of data were normally distributed and had the same changing trend, and in accordance with their formula, the erythrocyte ChA was selected as representative to fully simplify the analysis process.

### 3.4. The effects of sex, age, smoking, drinking and working duration on cholinesterase activity

The results in table 2 show that the cholinesterase activities of red blood cells were associated with gender, smoking and drinking in the exposure group (*p *< 0.05), whereas age and working duration had no effect (*p *> 0.05); gender, age, smoking and drinking had an effect on the ChA in the control group (*p *< 0.05). Cholinesterase activities had statistically significant differences between the two groups in different stratification of gender, age, smoking and drinking (*p *< 0.001). According to the segments of Chinese age, we regarded 40 years old as the boundary and divided age into the low age group and the high age group.

**Table 2.** The effects of sex, age, smoking, drinking and working duration on cholinesterase activity. p* indicates the comparisons of cholinesterase activity between the exposure group and the control group after stratification with two independent sample *t*-test; *p*^#^ represents the comparisons among the layers after stratification with one-way analysis of variance or two independent sample *t*-test.

	exposure		control			
variables	*n*	$\bar{x}\pm s$	*n*	$\bar{x}\pm s$	t*	p*
gender						
male	137	2.16 ± 0.54	54	3.42 ± 0.54	14.504	<0.001
female	43	1.87 ± 0.37	61	2.75 ± 0.58	9.351	<0.001
*t*^#^		−3.920		−6.333		
*p*^#^		<0.001		<0.001		
age						
≤40	53	2.01 ± 0.44	67	3.18 ± 0.59	12.332	<0.001
>40	127	2.12 ± 0.54	48	2.90 ± 0.70	6.921	<0.001
*t*^#^		1.426		−2.273		
*p*^#^		0.157		0.025		
smoking						
yes	63	2.24 ± 0.52	12	3.53 ± 0.42	8.037	<0.001
no	117	2.01 ± 0.50	103	3.01 ± 0.65	12.629	<0.001
*t*^#^		−2.995		−3.803		
*p*^#^		0.003		0.001		
drinking						
yes	16	2.39 ± 0.58	30	3.43 ± 0.53	6.102	<0.001
no	164	2.06 ± 0.50	85	2.93 ± 0.64	10.871	<0.001
*t*^#^		−2.483		−3.801		
*p*^#^		0.014		<0.001		
working duration						
<15	26	2.12 ± 0.47				
15∼30	117	2.09 ± 0.53				
>30	37	2.07 ± 0.52				
*F*^#^		0.066				
*p*^#^		0.936				

### 3.5. The relationship between genetic polymorphisms and cholinesterase activity

The genotype distribution for each genetic polymorphism locus did not deviate from the Hardy–Weinberg balance (*p *> 0.05), suggesting the control samples had representativeness. We regarded the wild homozygous genotype as a reference to analyse the differences of cholinesterase activities between the genotypes. As the wild homozygous genotype of the rs1625895 locus and the mutation homozygous genotype of the rs17878362 locus for gene *p53* were only one case, respectively, in the exposure group, they would merge with the heterozygous genotype directly. The relationships between genetic polymorphism and ChA are illustrated in table 3. The results show that there was a statistically significant difference in the ChA among the genotypes of the *p21* rs1801270 locus in the control group (*p *= 0.033), and there were no statistically significant differences among the genotypes of other loci (*p *> 0.05).

**Table 3.** The relationship between gene polymorphism and cholinesterase activity. p*, cholinesterase activity was compared among genotypes using one-way analysis of variance or two independent sample *t*-test after merging the genotypes. p**, the results between the two groups were compared using the Dunnett method.

		exposure			control	
SNPs	*n*	$\bar{x}\pm s$	p**		$\bar{x}\pm s$	p**
*p53* rs1042522						
CC	43	2.16 ± 0.49	ref^a^	23	3.13 ± 0.70	ref^a^
CG	73	2.00 ± 0.50	0.190	57	3.12 ± 0.68	0.999
GG	64	2.14 ± 0.55	0.962	35	2.92 ± 0.56	0.369
*F*(p*)		1.719(0.182)			1.181(0.311)	
*p53* rs17878362						
SS	162	2.10 ± 0.51		106	3.07 ± 0.65	
SL + LL	18	1.99 ± 0.58		9	2.95 ± 0.75	
*t*(p*)		0.825(0.411)			0.545(0.587)	
*p53* rs1625895						
GG	159	2.09 ± 0.51		106	3.07 ± 0.64	
AG + AA	21	2.12 ± 0.59		9	2.99 ± 0.79	
*t*(p*)		0.325 (0.746)			0.334 (0.739)	
*p21* rs1801270						
CC	61	2.14 ± 0.58	ref^a^	40	3.04 ± 0.67	ref^a^
CA	76	2.08 ± 0.52	0.310	48	3.22 ± 0.60	0.713
AA	42	2.06 ± 0.42	0.281	27	2.82 ± 0.66	0.646
*F*(p*)		0.380(0.684)			3.532(0.033)	
*p21* rs1059234						
CC	58	2.10 ± 0.56	ref^a^	40	3.07 ± 0.68	ref^a^
CT	78	2.07 ± 0.52	0.909	55	3.11 ± 0.62	0.940
TT	44	2.11 ± 0.45	0.999	20	2.92 ± 0.70	0.627
*F*(p*)		0.094(0.910)			0.614(0.543)	
*mdm2* rs3730485						
SS	11	2.24 ± 0.71	ref^a^	11	3.11 ± 0.60	ref^a^
SL	65	2.16 ± 0.51	0.761	38	2.96 ± 0.75	0.669
LL	103	2.03 ± 0.50	0.260	66	3.11 ± 0.60	1.000
*F*(p*)		1.806(0.167)			0.670 (0.514)	

^a^ref: The reference group for comparing different genotypes.

### 3.6. Effects of gene–gene and gene–environment interactions on cholinesterase activity

Table 4 lists the best models, prediction accuracies and cross-validation consistencies, and *p-*values were based on 1000 permutation replications. In the gene–gene interaction analysis, we have not found a significant gene–gene interaction model with the adjustment of gender, age, smoking, drinking and working duration (*p *> 0.05). However, according to the gene–environment interaction analysis after the adjustment of age and working duration, the results in row 7 show that *p53* rs1042522, *p53* rs1625895, *p21* rs1801270, *mdm2* rs3730485, sex, smoking and drinking had a potential interaction with ChA (*p *< 0.05), indicating that the ChA was the result of a combination of genes and environmental factors.

**Table 4.** Best models with covariate adjustment for cholinesterase activity data. *p-value based on 1000 permutation replications.

locus no.	best model	prediction accuracy	cross-validation consistency	p*
gene–gene interactions^a^				
1	*p21* rs1801270	0.4668	3/5	0.774
2	*p53* rs17878362*, p21* rs1801270	0.5387	2/5	0.220
3	*p53* rs1042522*, p21*rs1801270*, mdm2* rs3730485	0.4525	3/5	0.813
4	*p53* rs1042522*, p21* rs1801270*, p21* rs1059234*, mdm2* rs3730485	0.4190	4/5	0.941
5	*p53* rs1042522*, p53* rs1625895*, p21* rs1801270*, p21* rs1059234*,mdm2* rs3730485	0.4822	3/5	0.651
6	*p53* rs1042522*, p53* rs1625895*, p53* rs17878362*, p21*rs1801270*, p21* rs1059234*,mdm2* rs3730485	0.4736	5/5	0.711
gene–environment interactions^b^				
1	sex	0.6867	5/5	<0.001
2	*p53* rs1042522, sex	0.6867	3/5	<0.001
3	*p53* rs1042522*, p53* rs17878362, sex	0.7038	1/5	<0.001
4	*p53* rs1042522*, p21* rs1801270*, mdm2* rs3730485, sex	0.6246	2/5	0.006
5	*p53* rs1042522*, p21* rs1801270*, mdm2* rs3730485, sex, drinking	0.6347	2/5	0.001
6	*p53* rs1042522*, p21* rs1801270*, mdm2* rs3730485, sex, smoking, drinking	0.6437	3/5	0.003
7	*p53* rs1042522*, p53* rs1625895*, p21* rs1801270*, mdm2* rs3730485, sex, smoking, drinking	0.6235	2/5	0.006
8	*p53* rs1042522*, p53* rs1625895*, p21* rs1801270*, p21* rs1059234*, mdm2* rs3730485, sex, smoking, drinking	0.5573	5/5	0.182
9	*p53* rs1042522*, p53* rs1625895*, p53* rs17878362*, p21* rs1801270*, p21* rs1059234*, mdm2* rs3730485, sex, smoking, drinking	0.5540	5/5	0.160

^a^Adjusted for gender, age, smoking, drinking and working duration for gene–gene interaction analysis.

^b^Adjusted for age and working duration for gene–environment interaction analysis.

## 4. Discussion

In recent years, acute poisoning incidents caused by OPs have been reduced, while the chronic toxic effect on human health caused by long-term, low-dose exposure to OPs has attracted people's attention gradually [27,28]. Cell-cycle-regulating genes may regulate ChA in the body because these genes participate in cell damage regulation and apoptosis. There are possibly two main reasons for the changes in erythrocyte ChA. First, tissue homeostasis requires an orchestrated balance between cell proliferation, cellular senescence and cell death [29]. Cells proliferate through a cell cycle that is tightly regulated by cyclin-dependent kinase activities. Cellular senescence is a safeguard programme limiting the proliferative competence of cells in living organisms. Apoptosis eliminates unwanted cells by the coordinated activity of gene products that regulate and effect cell death. Therefore, the cell-cycle genes can affect the quantity and quality of peripheral red blood cells which could influence the ChA [30,31]. Second, the cell-cycle genes can affect chromosomal stability, and then affect the expression levels of cholinesterase-related genes, and thus affect the formation and degradation of cholinesterase. Previous studies have found that OP exposure can cause chromosomal DNA damage including telomere damage, DNA double- and single-strand breaks, chromosome aberration and micronucleus [6,9,32,33]. Therefore, this study investigated the effect of cell-cycle genes on ChA. The study is the first to explore the interaction between cell-cycle genes and environmental factors in regulating ChA through the GMDR method. The principal finding was that *p53* rs1042522, *p53* rs1625895, *p21* rs1801270, *mdm2* rs3730485, sex, smoking and drinking had a potential interaction with ChA (*p *< 0.05), indicating that the change in ChA was the result of a combination of genes and environmental factors.

Univariate analysis showed that cholinesterase activities in men, smokers and drinkers were higher than those of women, non-smokers and non-drinkers in the exposure or control group, respectively (*p *< 0.05), and ChA in the younger age group was significantly higher than that of the older age group in the control group (*p *< 0.001). Karasova *et al.* [34] found that ChA in smokers was higher than that in non-smokers (*p *< 0.05), which was consistent with our results in this study. This may be due to the components of tobacco smoke that stimulate the nervous system to increase the ChA, although further research still needs to address the specific mechanisms. However, a study from Arrieta *et al.* showed erythrocyte ChA had an association with age unlikely to be clinically significant [30]. We also found no interaction between age and other factors in the interactive analysis, suggesting that ChA was not affected by age.

Single-locus analyses showed there was a statistically significant difference in the ChA among the genotypes of the *p21* rs1801270 locus in the control group (*p *= 0.033), and there were no statistically significant differences among the genotypes of other loci (*p *> 0.05).This indicates that the interaction of multiple single nucleotide polymorphisms (SNPs) may function in ChA although a single SNP's function seems to be negligible in analysing each SNP separately. In brief, GMDR provides the possibility for the discovery of some micro-effect genes; otherwise, the significance of these genes will be lost when ignoring inherent gene–gene interactions.

However, the nature of the gene–environment interactions among these SNPs, sex, smoking and drinking is not clear. It may be due to the genetic variation of the cell-cycle genes that affects the quantity and quality of red blood cells, which are related to the erythrocyte ChA. However, the specific purpose of this work was to investigate the gene–gene and gene–environment interactions with the hypothesis that small single-factor effects could not be detected by single-locus studies. In this study, gene polymorphism had no effect on ChA. However, by the GMDR analyses, we further established interaction models among genes and environmental factors; a potential interaction was implicated by the significant four-locus model involving the three genes *p53*, *p21* and *mdm2* (*p *< 0.05). In the previous reports, *p53* is a tumour suppressor gene involved in the G_1_–S checkpoint and has the function of gene guarding. The *p21* gene is located in the downstream position of the *p53* gene in the signal path, and is a cyclin-dependent kinase inhibitor (CDKI); *p21* and *p53* constitute the G_1_–S checkpoints, so damaged cells stagnate in the G1 phase to reduce the formation of mutations and thus provide a tumour suppressor effect. At the same time, the *p53* expression caused by DNA damage is regulated negatively by the *mdm2* gene; *mdm2* is an important regulator of *p53* and has the function of degrading *p53*, and is also involved in tumour growth and metastasis [35].Vargas-Torres *et al*. [18] studied *p21* rs1801270 and rs1059234 genetic polymorphisms in 283 cases of cervical cancer patients and 189 cases of normal people; the results showed that mutant homozygous and heterozygous genotypes of the above two polymorphism loci were risk factors for cervical cancer. However, the results in this study indicated that the wild genotype (CC) and heterozygous genotype (AC) of *p21* rs1801270 may be protective factors for cholinesterase, which is not completely consistent with the studies mentioned above. Studies have shown that the 40 bp missing genotype (SS) for the *mdm2* rs3730485 locus may be a risk factor for uterine fibroids [36]; other studies also suggested that the SS type of this locus was a risk factor for hepatocellular carcinoma [37]. Although there was no report regarding the relationship between *p53* genetic polymorphisms and ChA in workers exposed to organophosphate pesticides, some researchers have studied the relationship between the three genetic polymorphism loci and micronucleus rate, and found no association between the three polymorphisms and micronucleus rate [38]. Again, we were also concerned regarding the potential biological mechanism under this interaction model among these SNPs and environmental factors in addition to the statistical significance. Therefore, in the next study we will plan to use the dual luciferase reporter gene assay to functionally verify the positive sites found in this study and to explain the effect of genetic variation on ChA from the perspective of molecular mechanisms.

The recently developed GMDR method provides covariate adjustment and is applicable to both dichotomous and continuous phenotypes to overcome the drawbacks of the MDR approach [39–41]. As presented in our results, the strength of interactions was different between with and without covariate adjustment, verifying that the loss of prediction ability may result from ignoring a covariate [39]. In our analyses, we employed gender, age, smoking, drinking and working duration as covariates.

In conclusion, our study has tested the association between three candidate genes and environmental factors in omethoate-exposure pollution based on single-locus and multilocus analyses. Our findings support the hypothesis that the SNPs from some of these candidate genes and environmental factors influence the pesticide-exposure reactions in an interactive manner. From the perspective of application and research, the significance of this discovery is: to screen susceptible populations and lay the theoretical foundation for the prevention and treatment of occupational diseases; and to discover the relevant genetic variation and provide clues for future study of the mechanism.

## Supplementary Material

Datasets

## Data accessibility

The datasets supporting this article have been uploaded as part of the electronic supplementary material. All data are available in the online electronic supplementary material.
